# Co‐Occurring Risk Factors for Tuberculosis Preventive Treatment Non‐Adherence Among People Living With HIV in Ethiopia: A Latent Class Analysis

**DOI:** 10.1111/tmi.70164

**Published:** 2026-05-14

**Authors:** Edao Sinba Etu, Jet Fransen, Biftu Geda, Kemal Ahmed Kuti, Anteneh Fikrie, Mark Spigt

**Affiliations:** ^1^ Department of Family Medicine CAPHRI School for Public Health and Primary Care, Maastricht University Maastricht the Netherlands; ^2^ School of Public Health, Shashamene Campus, Madda Walabu University Shashamene Ethiopia; ^3^ School of Public Health, Institute of Health, Bule Hora University Bule Hora Ethiopia

**Keywords:** adherence, Ethiopia, HIV, latent class analysis, tuberculosis preventive treatment

## Abstract

**Objective:**

Non‐adherence to tuberculosis preventive treatment (TPT) among people living with HIV (PLHIV) is a critical issue in Ethiopia and other Sub‐Saharan African countries, where both TB and HIV burdens are high. To reduce TPT non‐adherence among PLHIV, it is crucial to identify those at risk and implement targeted interventions. Therefore, the main objective is to identify subgroups based on co‐occurring risk factors for non‐adherence and examine their association with TPT adherence.

**Methods:**

A cross‐sectional study was conducted at selected health centres and hospitals in West Arsi, Ethiopia. Data from 390 PLHIV were collected through structured questionnaires administered via face‐to‐face interviews. Latent class analysis (LCA) was used to identify distinct subgroups, defined by risk factors for non‐adherence, among PLHIV. Maximum Likelihood adjustment method was used to predict adherence outcome based on the identified latent classes.

**Results:**

The overall prevalence rate of adherence in PLHIV was 60%. Two distinct latent classes emerged (prevalence rate noted): a “Lower risk of non‐adherence” class (63%), and a “Higher risk of non‐adherence” class (37%). PLHIV in the “Higher risk of non‐adherence” class endorsed higher probabilities of each of the considered risk factors, particularly regarding perceiving the relationship with the healthcare provider and distance to the health facility as barriers, and were nearly twice as likely to be non‐adherent compared to those in the “Lower risk of non‐adherence” class.

**Conclusions:**

The findings emphasize the importance of considering co‐occurring risk factors for non‐adherence and tailoring interventions accordingly. This study suggests specific practical implications: for example, screening tools assessing non‐adherence risk, health providers training programs and strengthening of community‐based primary care.

## 1 Background

Tuberculosis (TB) is the leading cause of death among people living with human immunodeficiency virus (PLHIV) [1]. PLHIV are 14 times more likely to develop active TB compared to those without HIV [1]. The co‐infection of HIV and TB results in a mutually reinforcing relationship, accelerating the progression of both diseases [2, 3]. Consequently, TB preventive treatment (TPT), which prevents latent tuberculosis infection (LTBI) from progressing to active TB, is crucial for PLHIV [4, 5]. As such, TPT is considered an essential component of the World Health Organization (WHO) End TB Strategy [4, 6].

Adherence to TPT is crucial for achieving optimal clinical outcomes [7, 8]. However, in Ethiopia and other Sub‐Saharan African countries, where there is a high burden of both TB and HIV, TPT non‐adherence poses a critical issue [9, 10]. TPT non‐adherence may result from people not feeling any signs or symptoms while still experiencing the burdens of taking medication, which is additionally challenging for PLHIV due to high pill burden and adverse effects. TPT non‐adherence can be understood as a multidimensional phenomenon shaped by a complex interplay of patient‐related, socioeconomic, treatment‐related, condition‐related, and health system/healthcare team‐related risk factors [11]. Hence, negative adherence outcomes can be attributed to the intersection or aggregation of risk factors rather than to any single risk factor [12]. Gaining insight into subgroups of PLHIV with co‐occurring risk factors, and determining which groups are associated with non‐adherence, could help identify PLHIV at risk for TPT non‐adherence and inform tailored interventions to improve adherence.

Latent class analysis (LCA) offers a person‐centred approach to analyze a complex set of observed characteristics (in this case “risk factors”) [13, 14]. It identifies unobserved subgroups, or classes, within the data that share similar patterns of characteristics. This approach classifies groups into those that are internally homogeneous (members of each subgroup showing similar patterns) and externally heterogeneous (there is clear differentiation between the groups) [13, 15]. Person‐centred approaches that account for the co‐occurrence of risk factors in individuals, such as LCA, are believed to achieve greater ecological validity than variable‐centred approaches, as they better highlight the important interplay among these factors, offering a more holistic understanding [16, 17]. LCA has been applied in various studies on adherence among PLHIV, but with a focus on antiretroviral therapy (ART) adherence [18, 19, 20, 21]. It has also been used in research on TB (preventive) treatment, examining aspects such as the performance of LTBI tests [22, 23], adherence tests [24], and healthcare utilization [25]. To our knowledge, LCA has not been used in studies on TPT adherence.

Given the complex interplay of factors influencing adherence, the limited resources faced by TB programs, and the impracticality of designing interventions tailored for each individual, LCA could provide a valuable approach that leads to more effective targeting to prevent non‐adherence [26]. Therefore, the objectives of this study were to: (1) identify and describe classes of PLHIV based on co‐occurring risk factors for non‐adherence using LCA. Factors considered include age, sex, religion, residence, educational status, knowledge about TPT, attitude towards TPT, socioeconomic status (SES), relationship with health care provider, and distance to health facility. (2) Examine whether the classes can predict TPT adherence outcomes. Since prior studies on risk classes of TPT non‐adherence are lacking, this study is exploratory and does not include specific hypotheses about the analysis of risk classes or the prediction of adherence outcomes based on these classes.

As of the 2025 zonal report, the total population of West Arsi Zone, including Shashemene City, is estimated to exceed 3.5 million. In 2018, the town was identified as one of the 200 towns with a high burden of HIV in Ethiopia and among the 48 towns in Oromia. As of December 2025, a total of 8557 people living with HIV in West Arsi and Shashemene city are currently receiving antiretroviral therapy. Preliminary result analysis from mega project estimated a tuberculosis prevalence of 15.07% (424/2813) while according to the 2025 final report, TPT coverage was 93% in West Arsi zone and 94% in Shashemene City.

### 1.1 Theoretical Framework

Adherence in the context of TB can be defined as “the extent to which the patient's history of therapeutic drug‐taking coincides with the prescribed treatment” [11]. Nonetheless, a universally accepted definition of TB medication adherence is lacking. To understand the interplay of factors shaping adherence, this study employs the WHO's Multidimensional Adherence Model (MAM), an ecological model that offers a holistic understanding of the various influences on adherence [11]. In this model, adherence is understood as a multidimensional phenomenon shaped by the interaction of five sets of factors, referred to as “dimensions.” These five dimensions encompass patient‐related factors, socioeconomic factors, treatment‐related factors, condition‐related factors, and healthcare system/healthcare team‐related factors. Patient‐related factors are just one dimension, emphasizing that adherence is not solely a patient‐driven problem. Socio‐economic factors include characteristics such as employment, income status, demographics (e.g., age; sex; marital status), stigma, educational status, and social support. Patient‐related factors include characteristics such as attitudes, beliefs, and knowledge. Treatment‐related factors include duration, regimen, and side effects. Condition‐related factors encompass characteristics such as comorbidities, severity of symptoms, and disease stage. Health system/healthcare team‐related factors include trust in health care providers, communication, quality of health services, distance, and healthcare insurance coverage. Across these dimensions, there are multiple factors that may vary among different populations, diseases, and settings [27]. The WHO's MAM provides a comprehensive lens through which this study explores and addresses TPT non‐adherence.

However, the selection of variables included in this study was mostly dictated by the availability of data within an existing questionnaire developed for a sub theme of TB–HIV co‐infection inside a larger mega project. This project divided into five major subthemes, each with several objectives that will be implemented in three phases over a 5‐year period. During phase I, data collection was carried out concurrently across sub‐themes, necessitating a concise questionnaire to reduce participant burden, given that participants were people living with HIV and had limited tolerance for lengthy interviews. As a result, it was not possible to incorporate all potentially important variables into each sub‐theme. Additionally, students participating in the project were supposed to develop their proposals and analyze data collected during their cohort period of the mega‐project due to funding constraints. Some variables (e.g., treatment regimen, disease stage, and side effects) were initially considered but dropped after the pilot study due to feasibility concerns, as many participants were unable to accurately report these clinical details, which could have compromised data quality.

## 2 Materials and Methods

### 2.1 Study Setting and Period

The study was conducted at public health institutions located in the West Arsi zone and Shashemene town, which are situated 252 km away from the capital city of Ethiopia. The study was conducted from February to August 15, 2023. The West Arsi zone is part of the Oromia regional state and the livelihoods of the people in the zone largely depend on agriculture. The zone consists of 12 woreda and two town administrations, covering an area of 540 sq. km. According to the 2025 Zonal report, the population of this zone was 2,972,534, with females comprising 51% and males making up the remaining population; the population of Shashemene city was 530,943, with females comprising 49.5%. Health services in the zone are provided by 7 hospitals and 102 health centres. Among these, 17 health centres and all hospitals offer TPT services at ART clinics. Shashemene town is the capital of the West Arsi zone and is one of the cities restructured as a Regiopolitan city along with three other cities in Oromia. The town is located at an altitude of 7° 12′ north and longitude 38° 36′ east. In 2018, the town was identified as one of the 200 towns with a high burden of HIV in Ethiopia and one of the 48 towns with a high burden of HIV in Oromia. As of December 2025, the town and West Arsi have 8557 people living with HIV who are currently receiving antiretroviral therapy, of whom 6160 (72%) are from Shashemene city. Shashemene town has one governmental comprehensive specialized referral hospital, one general hospital, six health centres, one private general hospital, and two private primary hospitals. Among these public health facilities, two hospitals and three health centres currently offer TPT services.

### 2.2 Participants and Procedures

A quantitative, facility‐based cross‐sectional study was conducted as part of an ongoing research project on the management of latent tuberculosis infection treatment among PLHIV in West Arsi, Ethiopia. Adults aged 18 years and older, diagnosed with HIV, who had initiated or completed TPT—either 6 months of daily isoniazid or 3 months of weekly rifapentine plus isoniazid (3HP) within the last month, following the introduction of the 3HP regimen in Ethiopia—were recruited during their (routine) appointments by ART clinic providers at health centres and hospitals in West Arsi.

A two‐stage sampling process was employed to select study participants and health facilities. Out of 26 public health facilities (6 hospital and 20 health centres) offering TPT services in West Arsi, five health centres and four hospitals were selected using simple random sampling. The first step in preparing the sampling frame involved preparing a list of potential study participants from each of the nine selected health facilities. The number of people living with HIV who have already initiated TPT in each facility was then taken into account to allocate the proportionate number of individuals was included in the sample from each facility and finally, participants within each facility were also randomly chosen. Data were collected using a structured questionnaire available in English, Oromo, and Amharic, administered through face‐to‐face interviews by 21 ART clinic providers. Data collection occurred after participants received the healthcare services they sought (i.e., exit interview) and after assessing their eligibility and willingness to participate, during their routine monthly follow‐up visits at the health facility, who had started or completed TPT within the previous month. This study falls under the sub theme of TB‐HIV co‐infection of a larger mega project. The total sample size determined for this sub theme was 564. For this study, however, there are no concrete guidelines for sample size for LCA models as of yet, but more than 300 is the most commonly used fit indices for mixture models. To ensure an appropriate sample size, more than 300 cases were included in the study, which aligns with commonly recommended requirements [28].

The data were collected from February 2024 through August 2024. TPT is routinely provided by ART clinic providers, and ART clinic providers received training from the research team on study objectives, interview techniques, and data management prior to data collection.

A pre‐test of the questionnaire was conducted 1 week prior to the commencement of the actual data collection at Kokosa General Hospital and Busa Health centre, which were not included in the main study. Based on the pilot findings, modifications were made to improve feasibility and data quality. For instance, variables such as treatment regimen, disease stage, and side effects were excluded, as participants were unable to accurately report these clinical details. The reliability of the questionnaire was assessed using internal consistency measures. Cronbach's alpha coefficients were computed for the entire instrument and for each KAP scale. The overall Cronbach's alpha for the study questionnaire score of 0.7 and above was considered acceptable. Prior to data collection, potential participants were informed about the study and verbal consent was obtained.

### 2.3 Measures

#### 2.3.1 Latent Class Indicators

Data on the 10 indicators were collected utilizing sections of a questionnaire adapted from the TPT Implementation Tools, developed by the U.S. President's Emergency Plan for AIDS Relief, which was modified to the local context (Additional file 1) [29]. TPT implementation tools were originally designed to assess implementation status at national level, which included questions such as: What is the status of TPT in this country?, Please list the barriers in this country to implementing TPT, Does this country currently perform adverse event monitoring for TPT? Etc. National‐ level data is the focus of these tools. Based on variables from intake and follow up records at healthcare facilities, including standardized formats of national forms and registers, pertinent components were tailored to the patient level in subnational setting for this study. The questionnaire was expanded to include patient level measures such as Morisky medication adherence Scale, wealth index questions, Knowledge, attitude and practice items, as well as institutional and healthcare provider‐related factors, in addition to implementation‐ related variables.

If necessary, the indicators were recategorized for the purpose of LCA. Age was measured in years at the time of questionnaire administration and categorized into four groups based on quartiles (≤ 28 years; 29–35 years; 36–42 years; ≥ 43 years). Additionally, sex (female; male), religion (Orthodox Christian; Muslim; Protestant), residence (Urban; Rural), and educational status (no formal education; primary school (1–6); secondary school (7–8); high school (9–12); diploma and above) were used to identify the latent classes. Educational status was dichotomized into no formal education and formal education. Knowledge about TPT was measured using seven items (e.g., “Are you aware of the purpose behind initiating use of TPT medication?”) with responses coded as “yes” = 1 or “no” = 0. Knowledge was dichotomized based on the frequencies of “yes” responses, with “Poor knowledge” for participants who answered “yes” between one and five times, and “Good knowledge” for participants who answered “yes” six or seven times. Attitude towards TPT was assessed with 12 items using a five‐point scale (strongly agree = 5; agree = 4; neutral = 3; disagree = 2; strongly disagree = 1) (e.g., “Latent TB treatment has serious negative consequences on my life”). Attitude was dichotomized based on the median total score: poor attitude (total score ≤ 49) and good attitude (total score ≥ 50). The SES index was measured using 25 asset items (e.g., “Where do you live/dwelling?”) and derived through principal component analysis, as proposed by Filmer and Pritchett [30] and Vyas and Kumanarayake [31]. The asset items for the index were based on the standard SES index described by Filmer and Pritchett [30] and the local context. The items included in the final wealth index were: living/sanitation conditions (water source, cooking fuel source, type of toilet), ownership of durable assets (electric mitad, television, radio), financial access (bank account or microfinance saving account), ownership of productive assets (livestock, farm animals, and agricultural land), access to social support (safety net programs, enrollment in community‐based health insurance). The first principal component, which explained 25.4% of the variance, was used to determine weighting factors for each included item and to define the SES index. The continuous SES index was disaggregated into quintiles, reflecting low, middle and high SES. Additionally, intuitional factors (barrier or not a barrier) and relationship with healthcare provider or healthcare provider‐related characteristics (barrier or not a barrier) were used to identify the latent classes. Respondents were given a list of potential barriers and asked to rate each barrier using a 3‐point scale (‘not a barrier’, ‘somewhat a barrier’, ‘important barrier’). Total scores and averages were determined from all of the individuals' responses. The mean score was used to categorize the participants into two groups: “Important barrier” and “not a barrier”.

#### 2.3.2 Outcome Variable

TPT adherence was assessed using the eight‐item Morisky Medication Adherence Scale (MMAS‐8), a questionnaire designed to measure medication adherence based on patients' self‐reported behaviour over a brief recall period (usually the preceding 7 days) (Additional file 1) [32]. This questionnaire measures adherence through seven yes‐or‐no items (e.g., “When you travel or leave home, do you sometimes forget to bring along your medicine?” [yes = 0]; “Did you take all your medicines yesterday?” [yes = 1]) and one item asking, “How often do you have difficulty remembering to take all your medicine?” on a 5‐point scale (never/rarely = 1; occasionally = 0; sometimes = 0; usually = 0; all the time = 0). Scores on the MMAS‐8 range from zero to eight, with a score below six indicating “poor” adherence (non‐adherence) and a score of six or above indicating “good enough” adherence (adherence) [32].

### 2.4 Statistical Analysis

Descriptive analyses were conducted using SPSS version 28.0.1.1 (IBM Corporation, Armonk, NY) to summarize the characteristics of the sample and provide context for the LCA. LCA uses multiple indicators to divide a population into underlying homogeneous classes [13]. To identify these latent classes based on 10 indicators—risk factors for non‐adherence—and to examine the association between these latent classes and adherence, a three‐step analysis was performed using LatentGOLD version 6.0 (Statistical Innovations, Arlington, MA). The steps were as follows: in step one, determining an optimal latent class model using the 10 indicators; in step two, classifying individuals into the latent classes based on their posterior class membership probabilities from step 1; and in the third step, the relationship between the latent class membership and an outcome variable (distal outcome) was examined using Maximum Likelihood (ML) correction approach that adjusts for classification errors/uncertainty introduced in step two by integrating posterior class probabilities, hence minimizing bias caused by misclassification [33, 34].

LCA was used to identify and describe latent classes based on 10 nominal and ordinal indicators: age, sex, religion, residence, educational status, knowledge about TPT, attitude towards TPT, SES index, distance to healthcare facility, and relationship with healthcare provider. Models with 1–4 classes were considered. For each class solution, LCA produces two sets of parameters. The first parameter represents the probability of membership in each latent class. The second parameter provides the conditional probability (item‐response probability) that an individual in a certain class will endorse specific responses (e.g., “Poor knowledge” or “barrier”) for each indicator. These parameters were used to interpret and label the latent classes.

The selection of the optimal model was based on penalized fit criteria (Bayesian Information Criterion (BIC), Akaike Information Criterion (AIC), and the modified AIC (AIC3)), bootstrapped likelihood ratio significance testing (BLRT), entropy, and model stability and interpretability [13, 35]. Lower values for BIC, AIC, and AIC3 indicate a better balance between model fit and parsimony. Since AIC tends to overfit while BIC favours a more parsimonious model, BIC was given more weight in the model selection process. Models for which the BLRT is significant (*p* < 0.05) indicate that the model fits significantly better than the model with one fewer class; non‐significant BLRT values suggest that an additional class is unnecessary. Conversely, high entropy values indicate better classification utility and class separation. Additionally, the assumption of local independence—the concept that variables within a latent class are independent of each other—was checked based on the bivariate residuals (BVRs). BVRs larger than 3.84 indicate correlations between indicator pairs that are not adequately explained by the model [34]. Missing values for any indicator were included in the analysis using ML estimation. Model identification was validated with 160 sets of random starting values for all candidate models.

The association between latent class membership and the outcome variable was estimated using an adjusted three‐step approach recommended for categorical variables [36]. The ML adjustment approach adjusts for classification uncertainty by factoring in posterior class membership probability and the related misclassification matrix while estimating class‐specific outcome parameters (means and variances by standard ML). The relationship between latent class membership and distal outcome was assessed using a global Wald test of equality of class‐specific outcome parameters across classes. A significant Wald test (*p* < 0.05) shows that the distal outcome differs between latent classes.

## 3 Results

### 3.1 Descriptive Statistics

The sample consisted of 390 participants who initiated or completed TPT. Of the 390 participants, 158 (40.5%) displayed non‐adherence. Table 1 presents the descriptive characteristics of the sample, including variables with missing values. Knowledge about TPT had missing values for 71 (18.2%) participants (Table 1).

**TABLE 1:** Descriptive characteristics of participants (*N* = 390).

Variables	Categories	*N*	%
Latent Class Indicators			
Age	≤ 28 years	105	27
	29–35 years	97	25
	36–42 years	94	24
	≥ 43 years	94	24
Sex	Female	234	60
	Male	156	40
Religion	Orthodox Christian	165	42
	Muslim	134	35
	Protestant	91	23
Residence	Urban	341	87
	Rural	49	13
Educational status	No formal education	90	23
	Formal education	300	77
Knowledge about TPT	Poor knowledge	71	18
	Good knowledge	248	64
	Missing	71	18
Attitude towards TPT	Poor attitude	205	52
	Good attitude	185	48
SES	Low	134	34
	Middle	122	31
	High	134	34
Distance to HF	Barrier	141	36
	Not a barrier	249	64
Relationship HCP	Barrier	57	15
	Not a barrier	333	85
Outcome variable			
Level of adherence	Non‐adherence	158	40
	Adherence	232	60

Abbreviations: HCP, health care provider; HF, health facility; SES, socioeconomic status.

### 3.2 Model Selection

The initial estimation showed substantial BVRs for certain pairs of indicators. To account for the significant overlap between some indicators, three indicators (sex, residence, and SES) were serially eliminated. Additionally, within‐class associations between attitude towards TPT and knowledge about TPT were allowed by adding their associated direct effect parameter. These adjustments reduced the maximum BVR from 17.8 to 3.4 in the 2‐class model.

Model selection was based on a pre‐specified hierarchical framework. The Bayesian Information Criterion (BIC) was used as the primary criterion, with lower values indicating better model fit while penalizing model complexity. The bootstrap likelihood ratio test (BLRT) was used as a secondary criterion to assess whether additional classes significantly improved model fit. Classification quality indicators, including entropy and classification error, were also considered. The Akaike Information Criterion (AIC) was considered supportive but not decisive, as it is known to favour more complex models. Based on these criteria, the two‐class model was selected as the optimal solution.

Model fit information and selection criteria for the latent class models are presented in Table 2. Models with 1 to 4 classes were compared to determine the optimal model. Although the AIC continued to decrease with the addition of a third class, both the BIC and AIC3 favoured the 2‐class model. The BLRT suggested an improved fit up to 3 classes (significant for 2 vs. 1 class, but not for 3 vs. 2 classes). This indicates that additional classes did not need to be considered. Therefore, our study selected the 2‐class model for interpretation and further analysis, with a moderate entropy (0.77) and lowest classification error (0.77). The relatively high entropy R^2^ value (i.e., > 0.80) and low estimated proportion of classification errors suggest adequate classification quality in the 2‐class model. The model showed sufficient class separation, high entropy, and good stability, indicating that the identified latent classes were sufficiently separated, individuals were categorized with high certainty, and the solution was robust and reproducible.

**TABLE 2:** Model fit information and selection criteria.

Classes	LL	BIC	AIC	AIC3	BLRT	Npar	Max. BVR	Class error	Entropy R^2^
1	−2012.45	4090.52	4046.90	4057.90		11	82.01	0.000	1.00
**2**	**−1933.82**	**3986.98**	**3907.66**	**3927.66**	**0.00**	**20**	**3.39**	**0.046**	**0.77**
3	−1923.76	4020.54	3905.52	3934.52	0.15	29	2.87	0.053	0.79
4	−1915.13	4056.97	3906.26	3944.26	0.31	38	1.64	0.096	0.74

*Note:* Bold values indicates selected class model for interpretation and further analysis.

Abbreviations: AIC, Akaike information criterion; AIC3, AIC with penalty 3; BIC, Bayesian information criterion; BLRT, bootstrapped likelihood ratio test; LL, Log likelihood.

### 3.3 Description of the Latent Classes

Table 3 presents the parameter estimates from the selected LCA model. Class one, representing approximately 63% of the sample and labelled “Lower risk of non‐adherence,” consisted of PLHIV with relatively low frequencies of factors associated with non‐adherence. Specifically, 61% reported being younger than 36 years, 29% identified as Muslim, 16% had no formal education, 15% had poor knowledge, and 35% had a poor attitude. Additionally, 4% considered the distance to the health facility a barrier, and 1% regarded the relationship with the healthcare provider as a barrier. Class 2, accounting for approximately 37% of the sample and labelled “Higher risk of non‐adherence,” comprised PLHIV with higher frequencies of factors associated with non‐adherence. In this class, 36% reported being younger than 36 years, 44% identified as Muslim, 35% had no formal education, 36% had poor knowledge, and 60% had a poor attitude. Moreover, 92% considered the distance to the health facility a barrier, and 38% regarded the relationship with the healthcare provider as a barrier (Table 3).

**TABLE 3:** Parameter estimates for the 2‐class model (*N* = 390).

		Latent class			
	Overall sample	Class 1, Lower risk of non‐adherence	Class 2, Higher risk of non‐adherence	Wald	*R* ^2^
		Latent class membership probabilities			
		0.63	0.37		

Indicators		Item‐response probabilities			
Age				19.74***	0.07
≤ 28 years	0.27	0.34	0.15		
29–35 years	0.25	0.27	0.21		
36–42 years	0.24	0.22	0.28		
≥ 43 years	0.24	0.17	0.36		
Religion				7.72*	0.01
Orthodox Christian	0.42	0.47	0.34		
Muslim	0.35	0.29	0.44		
Protestant	0.23	0.24	0.22		
Educational status				13.18***	0.05
No formal education	0.23	0.16	0.35		
Formal education	0.77	0.84	0.65		
Knowledge about TPT				7.47**	0.06
Poor knowledge	0.23	0.15	0.36		
Good knowledge	0.77	0.85	0.64		
Attitude towards TPT				11.26***	0.06
Poor attitude	0.44	0.35	0.60		
Good attitude	0.56	0.65	0.40		
Distance to HF				14.33***	0.79
Barrier	0.36	0.04	0.92		
Not a barrier	0.64	0.96	0.08		
Relationship HCP				10.95***	0.26
Barrier	0.15	0.01	0.38		
Not a barrier	0.85	0.99	0.62		

*Note:* Latent class membership probabilities indicate the proportion of PLHIV in each latent class. Item‐response probabilities represent proportions of PLHIV in a particular class endorsing an item. Wald test is used to test if the indicator discriminates between the classes. R^2^ reflects the communality of the indicator. *p < 0.05; **p < 0.01; ***p < 0.001.

Abbreviations: HCP, health care provider; HF, health facility.

### 3.4 Association of the Latent Classes With Outcome Variable

The association between these two latent classes and the outcome variable (adherence vs. non‐adherence) was examined. Table 4 presents the proportions of PLHIV experiencing the outcome, conditional on latent class membership. There was a highly statistically significant difference in adherence between the “Higher risk of non‐adherence” and “Lower risk of non‐adherence” class (*p* < 0.001). Membership in class 2 (“Higher risk of non‐adherence”) was associated with a higher proportion (0.59) of TPT non‐adherence relative to class 1 (“Lower risk of non‐adherence”).

**TABLE 4:** Class specific proportions of PLHIV with adherence versus non‐adherence.

Outcome variable	Overall sample	Class 1, Lower risk of non‐adherence (63%)	Class 2, Higher risk of non‐adherence (37%)	Wald
		Item‐response probabilities		
Level of adherence				26.50***
Non‐adherence	0.41	0.30	0.59	
Adherence	0.59	0.70	0.41	

*Note:* Item‐response probabilities indicate proportions of PLHIV in a particular class endorsing an item (adherence vs. non‐adherence). Wald statistic tests whether there is a significant difference in level of adherence between the classes. ***p < 0.001.

## 4 Discussion

In this study among PLHIV in West Arsi, Ethiopia, the overall adherence prevalence rate was 60%. Using LCA, two subgroups were identified based on co‐occurring risk factors for non‐adherence: a “Lower risk of non‐adherence” group, comprising 63% of the PLHIV, and a “Higher risk of non‐adherence” group, comprising 37% of the PLHIV. PLHIV in the higher risk group were nearly twice as likely to be TPT non‐adherent, highlighting the importance of understanding the specific factors affecting this group. This “Higher risk of non‐adherence” group was characterized by increased age, being Muslim, poor knowledge about TPT, a negative attitude towards TPT, lack of formal education, and perceiving the relationship with healthcare provider and distance to health facility as barriers. Particularly, the latter two proved to be distinguishing factors that strongly contributed to the “Higher risk of non‐adherence” group. The findings underscore the importance of targeted interventions to reduce the impact of non‐adherence among PLHIV, especially in the high‐risk group.

The overall prevalence rate of TPT adherence in this study was 60%. Nevertheless, studies have shown that TPT adherence rates vary significantly, reporting both higher and lower adherence rates. For example, a study in Uganda [37] reported an adherence rate of 81% and a study in Brazil [38] reported an adherence rate of 53%. Studies in South Africa [39], Ethiopia [40] and the United States [41] revealed adherence rates of 69%, 90.3%, and 40%, respectively. These differences can partly be explained by the various strategies used to assess TPT adherence. However, the differences also indicate variations in underlying risk factors depending on the setting and population. This underscores the necessity for interventions tailored to the specific risk factors in each context.

Studies have demonstrated the role of attitude, knowledge, age, religion, relationship with the healthcare provider, distance to health facilities, and educational status on adherence among PLHIV [42, 43, 44, 45, 46, 47, 48]. A critical advancement of the current study is that, instead of controlling for the independent effects of these factors, the combined and synergistic effects of several factors on TPT adherence were explored [16]. The LCA revealed two distinct subgroups based on co‐occurring factors for non‐adherence. The “Lower risk of non‐adherence” group, comprising 63% of the PLHIV, exhibits a combination of protective factors that collectively reduce the risk of non‐adherence among PLHIV. Individuals in this subgroup were almost twice as likely to be adherent compared to those in the “Higher risk of non‐adherence” group. Importantly, PLHIV in this group can provide peer support, sharing their experiences with those in the higher‐risk group to improve adherence rates by helping to build trust in healthcare and enhancing knowledge and attitudes towards TPT [49].

In contrast, the “Higher risk of non‐adherence” group is characterized by its higher probability of endorsing all seven risk factors. These findings support studies emphasizing that TPT adherence is a complex phenomenon attributed to the intersection or accumulation of risk factors rather than a single one [43, 44]. Moreover, the findings indicate that perceiving distance to healthcare—both in a physical (distance to the health facility) and a relational (the relationship with the healthcare provider) sense—strongly contributes to the “Higher risk of non‐adherence” group. This shows that, although socioeconomic and individual factors increase the risk of non‐adherence, adherence is not isolated from the broader context in which healthcare system and team‐related factors also play an important role. Thus, the findings imply that interventions aimed at improving TPT adherence are crucial for PLHIV with multiple risk factors, especially for those who also perceive distance to healthcare. Given that PLHIV in the “Higher risk of non‐adherence” group are nearly twice as likely to be non‐adherent, treatment programs could conduct screenings for similar risk factors before initiating TPT among PLHIV to assess the risk of non‐adherence. In Ethiopia, TPT services are provided as part of HIV care for PLHIV [50], meaning providers of HIV care may be well‐suited to identify those at risk of non‐adherence by using a screening tool focused on relevant risk factors.

The distribution of protective and risk factors among the subgroups offers valuable insights for intervention design. As such, non‐adherence prevention interventions for PLHIV should not focus on individual risk factors, but rather adopt a more holistic approach. Interventions that address barriers related to the relationship with healthcare providers and distance to healthcare may be most effective in supporting adherence. Specifically, interventions could focus on improving counselling and communication between healthcare providers and patients through appropriate training, as well as on strengthening community‐based primary care. This could potentially bridge the gap between healthcare and PLHIV. In Senegal, a cluster RCT demonstrated that a multitargeted intervention including training healthcare providers to enhance counselling and communication, as well as decentralizing treatment and involving community health workers (CHWs), led to improved adherence to TB treatment in a low‐resource setting [51].

Although Ethiopia has made notable progress in community‐based primary healthcare through the Health Extension Program in recent years [52], the findings suggest that healthcare‐related factors are associated with TPT adherence. The findings from this study may provide useful insight for programmatic consideration. Previous research has demonstrated that strengthening community‐based primary healthcare, such as enhancing the role of community health workers (CHWs) and boosting access to HIV care and TPT services at the community or home level, can help with adherence in low‐resource settings [53, 54, 55, 56, 57]. For example, community based intervention in Ethiopia have shown encouraging outcomes in improving TPPT adherence among children [58]. These measures may also have an indirect impact on associated factors, such as patient knowledge and attitudes, by improving education and support. Similarly, improving health provider communication and counselling may increase patient engagement with care. Nonetheless, these possible mechanisms should be viewed with caution, and more studies with a larger range of variables are required to better understand the causal pathways impacting TPT adherence.

The study has limitations. Assessing self‐reported TPT adherence poses challenges, including recall and social desirability bias, which, despite the interview‐based setting, may have led to underreporting of non‐adherence [8]. Risk factors may also be influenced by social desirability bias; for instance, perceiving the relationship with the healthcare provider as a barrier may be underreported, since the interviews were conducted by ART clinic providers. Additionally, the questionnaire used to collect data on risk factors for TPT non‐adherence presents a limitation. Since it was not specifically designed for this study, the selection of risk factors was restricted to those included in the questionnaire. As a result, several important factors—such as stigma, lack of trust in the healthcare system, social support, and costs, which are often linked to adherence—were excluded [44, 48]. Evaluating the selection of risk factors through the WHO's MAM reveals that key factors related to treatment, condition, and health system/health care team were largely overlooked, placing too much emphasis on patient‐related factors and the community or environment. Furthermore, there are no specific guidelines for sample size in LCA models, which means the sample size in this study may not have been large enough. However, the models were well‐identified, and the emerging classes were relevant. While Latent GOLD's three‐step approach used to assess the relationship between the latent classes and the distal result (TPT adherence), we acknowledge that external validation using independent data would improve the findings' generalizability and recommend it for future research. Finally, the geographical focus of the study on the West Arsi zone in Ethiopia may limit the generalizability of the findings to other regions or countries.

Since there is no gold standard for assessing medication adherence [8], future research should employ a combination of methods—both direct and indirect—allowing one method to serve as a reference standard for another, helping to prevent bias. Additionally, future studies should reconsider the choice of risk factors; by including key factors from all five dimensions, researchers could achieve more effective identification of risk groups and further inform targeted interventions.

## 5 Conclusions

To reduce TPT non‐adherence among PLHIV, identifying PLHIV at risk for non‐adherence and implementing targeted interventions is crucial. This study identified two distinct non‐adherence risk subgroups based on co‐occurring risk factors: a “Lower risk of non‐adherence” group, exhibiting consistently protective factors, and a “Higher risk of non‐adherence” group, facing multiple risk factors. PLHIV in the “Higher risk of non‐adherence” group were more likely to experience negative adherence outcomes compared to those in the “Lower risk of non‐adherence” group. The findings highlight the importance of considering co‐occurring risk factors to adherence and tailoring interventions accordingly. Based on this, healthcare providers should conduct screenings for non‐adherence risk, improve their communication and counselling skills, and offer comprehensive community‐based primary care. Policymakers should further support and strengthen community‐based primary care programs. Additionally, future studies should reconsider the risk factors used to identify the subgroups to more effectively map PLHIV at risk. Hopefully, these findings will lead to improved screening methods and intervention strategies, ultimately reducing the TB burden by fostering TPT adherence among PLHIV.

## Funding

This work is supported by Madda Walabu University under the megaproject titled “Universal Test and Treat Strategy of HIV: Outcomes, Co‐morbidities, and Implications for Intervention,” with a sub‐theme on the “Management of Latent Tuberculosis Infection Treatment among People Living with HIV in West Arsi, Ethiopia.”

## Ethics Statement

This study was approved by the Institutional Research Ethics Review Committee of Madda Walabu University (reference number: IRERC/046/2023). Verbal consent was obtained from all participants. Before giving informed consent, participants were informed about the study by trained data collectors. The data were collected and analyzed anonymously.

## Consent

The authors have nothing to report.

## Conflicts of Interest

The authors declare no conflicts of interest.

## Supporting information


**Appendix S1:** English version questionnaire TPT implementation status.
**Appendix S2:** English version MMAS‐8.

## Data Availability

The dataset generated and analyzed during the current study is available in the figshare repository, https://doi.org/10.6084/m9.figshare.27868218.v1.
