# Supplementary material for: Co‐Occurring Risk Factors for Tuberculosis Preventive Treatment Non‐Adherence Among People Living With HIV in Ethiopia: A Latent Class Analysis
Source: Trop Med Int Health. 2026 May 14;31(9):1250–60. doi: 10.1111/tmi.70164 (PMC13533639; doi:10.1111/tmi.70164)
Supplement: Supplementary file 1 [file TMI-31-1250-s001.docx]

## **Additional file 1.**

## **Appendix S1.** English version questionnaire TPT Implementation status.

|  | **Identification** | | | | | | | | |  | |  | |  |
| --- | --- | --- | --- | --- | --- | --- | --- | --- | --- | --- | --- | --- | --- | --- |
|  | SD01 | Write the name of the health facility | |  | | | | | |  | |  | |  |
|  | SD02 | Do you live in the urban or the rural? | | 0. Urban 1. Rural | | | | | |  | |  | |  |
|  | SD03 | Write resident district of patient | |  | | | | | |  | |  | |  |
|  | SD04 | Write name of permanent resident district name of patient | |  | | | | | |  | |  | |  |
|  | SD05 | Write resident kebele of patient | |  | | | | | |  | |  | |  |
|  | SD06 | Patient ART unique number | |  | | | | | |  | |  | |  |
|  | SD07 | Do you have your own cell phone number? | | Yes  No | | | | | |  | |  | |  |
|  | SD08 | If yes, may we please have your contact number? | |  | | | | | |  | |  | |  |
|  | SD09 | If no, may we have your neighbour’s contact number so that we can reach out to you for any future communication? | |  | | | | | |  | |  | |  |
|  | **i. Individual related factors: Socio demographic factors** | | | | | | | | |  | |  | |  |
|  | **N^O^** | **Question** | | **Response** | | | | **coding** | |  | | |  |  |
|  | SD10 | How old are you? | | ____years | | | |  | |  | | |  |  |
|  | SD10 | Sex | | Female  Male | | | | 0  1 | |  | | |  |  |
|  | SD11 | Current marital status | | Single  Married  Divorced  Widowed  Other(specify) _______ | | | | 0  1  2  3  4 | |  | | |  |  |
|  | SD12 | Religion | | Orthodox christens  Muslim  Protestant  Other(specify)_______ | | | | 0  1  2  3 | |  | | |  |  |
|  | SD13 | Ethnic group | | Oromo  Amhara  Welayta  Other(specify)__________ | | | | 0  1  2  3 | |  | | |  |  |
|  | SD14 | Education status | | No formal education  Primary school (1-6)  Secondary school (7-8)  Highschool (9-12)  Diploma and above | | | | 0  1  2  3  4 | |  | | |  |  |
|  | SD15 | Occupation | | Farmer | | | | 0 | |  | | |  |  |
|  |  |  |  | Student | | | | 1 | |  | | |  |  |
|  |  |  |  | Housewife | | | | 2 | |  | | |  |  |
|  |  |  |  | Merchant | | | | 3 | |  | | |  |  |
|  |  |  |  | Governmental /non-governmental employee | | | | 4 | |  | | |  |  |
|  |  |  |  | Daily labourer | | | | 5 | |  | | |  |  |
|  |  |  |  | Other(specify)_______ | | | | 6 | |  | | |  |  |
|  | **SD16** | **Assets** | | | | | | | |  | |  | |  |
|  | 1 | What is your main source of water? | | Pipe water  River/lake  Bore hole/well water  Other(specify)_______ | | | | 0  1  2  3 | |  | | |  |  |
|  | 2 | What kind of toilet do you have? | | Pit latrine  Pour flush latrine  Other(specify)________ | | | | 0  1  2 | |  | | |  |  |
|  | 3 | What type of fuel/energy does your HH commonly use? | | Electricity  Wood or charcoal  Biogas  Other(specify)___________ | | | | 0  1  2  3 | |  | | |  |  |
|  | 4 | Where do you live/dwelling? | | Owned house  Free of charge/subsidized  Rented from kebele/agency/employer  Rented from individual  On street  Hotel  Other(specify) _______ | | | | 0  1  2  3  4  5  6 | |  | | |  |  |
|  | 5 | Does your HH have the following items? | | Television Yes  No  Radio Yes  No  Electricity Yes  No  Electric mitad Yes  No  Sofa/Arabian Majilis Yes  No  Refrigerator Yes  No  Other(specify) _______ | | | | 1  0  1  0  1  0  1  0  1  0  1  0 | |  | | |  |  |
|  | 6 | Does any of your HH members own? | | Animal drawn cart Yes  No | | | | 1  0 | |  | | |  |  |
|  |  |  |  | Phone Yes  No | | | | 1  0 | |  | | |  |  |
|  |  |  |  | Bicycle Yes  No | | | | 1  0 | |  | | |  |  |
|  |  |  |  | Motorbike Yes  No | | | | 1  0 | |  | | |  |  |
|  |  |  |  | Bajaj/ car/track Yes  No | | | | 1  0 | |  | | |  |  |
|  |  |  |  | Other(specify) _______ | | | |  | |  | | |  |  |
|  | 7 | Does any member of HH have bank account or microfinance saving account? | | Yes  No | | | | 1  0 | |  | | |  |  |
|  | 8 | Is your HH receiving cash or food from safety net program? | | Yes  No | | | | 1  0 | |  | | |  |  |
|  | 9 | Is your HH enrolled in CBHI? | | Yes  No | | | | 1  0 | |  | | |  |  |
|  | 10 | Does your HH own any livestock, herds, farm animals, poultry farm or beehives? | | Yes  No | | | | 1  0 | |  | | |  |  |
|  | 11 | If yes, how many livestock, herds, farm animals, poultry farm or beehives does this HH own? | | Milk cow/ bulls________  Horse/donkey_______  Goat/sheep______  Chickens or other poultry _____  Beehives________  Other(specify)_______ | | | |  | |  | | |  |  |
|  | 12 | Does any member of this HH own agricultural land? | | Yes  No | | | | 1  0 | |  | | |  |  |
|  | 13 | If yes, how many hectares of land do your HH own? | | ------------------------------ | | | |  | |  | | |  |  |
|  |  | **ii. PLHIV who have already initiated TPT: knowledge toward latent TB infection treatment** | | | | | | | |  | |  | |  |
|  | K01 | Have you ever heard about latent TB infection | | Yes  No | | | | 1  0 | |  | | |  |  |
|  | K02 | Have you ever heard about latent TB infection treatment? | | Yes  No | | | | 1  0 | |  | | |  |  |
|  |  | If yes, how did you hear it? | | Media (newspapers/magazines, radio, and television)  Health workers  Family, friends and colleagues  Other(specify)_______ | | | | 0  1  2  3 | |  | | |  |  |
|  | K03 | Are you aware of the purpose behind initiating use of TPT medication? | | Yes  No | | | | 1  0 | |  | | |  |  |
|  |  | If yes, can you explain to me the reason you started using TPT? | | Medication is sick given to people with TB infection (latent TB) to prevent them from getting sick  I did use the medication, but I am unsure of the reason or intention behind starting its use  Other(specify)_______ | | | | 0  1  2 | |  | | |  |  |
|  | K04 | Do you know how to take TPT medication? | | Yes  No | | | | 1  0 | |  | | |  |  |
|  |  | If yes, how frequently did you take medication each week? Tick all possible answers | | Every day of the week  Once per week  twice per week  Three times per week  Other(specify)_______ | | | | 0  1  2  3  4 | |  | | |  |  |
|  | K05 | Do you know the common symptoms that may occur as side effects when taking TPT medication for latent tuberculosis infection? | | Yes  No | | | | 1  0 | |  | | |  |  |
|  |  | If yes, can you tell me them pls? | | Urine, saliva, tears, or sweat to appear an orange red colour  Fatigue  Jaundice  Nausea/vomiting/abdominal pain Itching  Neuropathy  I don’t remember  Other(specify)_______ | | | | 0  1  2  3  4  5  6 | |  | | |  |  |
|  | K06 | It is ok to take TPT with other HIV medicines | | Yes  No | | | | 1  0 | |  | | |  |  |
|  | K07 | Do you know the signs indicating that you should discontinue the use of TPT medication and consult a healthcare professional? | | Yes  No | | | | 1  0 | |  | | |  |  |
|  |  | If yes, list symptoms you may know | | Less or no appetite for food  Severe tiredness or feeling particularly unwell  An upset stomach or stomach cramps  Nausea or vomiting  Severe diarrhoea or dark-coloured urine or light stool  Skin rash or itching  Yellowing skin or eyes  Tingling or numbness in hands or feet/legs  Dizzy or lightheaded when sitting, standing, or lying down  Flu-like symptoms  Fevers or chills  Bruises or red and purple spots on your skin that you cannot explain  I don’t remember  Others, specify | | | | 0  1  2  3  4  5  6  7  8  9  10  11  12  13 | |  | | |  |  |
|  |  | If your response is no for above question, what do you do if you have a concern? Tick all possible answers | | I tell my doctor or nurse if I have questions or concerns  I tell my doctor/nurse about all other medicines, supplements or herbs I am taking  I discuss with my doctor any alcohol use  I stop medication without informing anyone  I do nothing | | | | 0  1  2  3  4 | |  | | |  |  |
|  |  | **iii. PLHIV who have already initiated TPT: attitude toward latent TB infection treatment. Now, I would like to ask about your attitude on LTBI treatment. Please rate the following statements on a scale of one to five** | | | | | | | |  | |  | |  |
|  |  |  | | **Strongly Disagree** | **Disagree** | | **Neutral** | | **Agree** | **Strongly agree** | |  | |  |
|  | AT01 | Latent TB infection treatment has severe side effect | | 5 | 4 | | 3 | | 2 | 1 | |  | |  |
|  | AT02 | I would rather die than taking latent TB infection treatment | | 5 | 4 | | 3 | | 2 | 1 | |  | |  |
|  | AT03 | Latent TB treatment has serious negative consequences on my life | | 5 | 4 | | 3 | | 2 | 1 | |  | |  |
|  | AT04 | Latent TB infection treatment is extremely harmful | | 5 | 4 | | 3 | | 2 | 1 | |  | |  |
|  | AT05 | I don’t like latent TB infection treatment because it causes my urine, saliva, tears, or sweat to appear an orange red colour | | 5 | 4 | | 3 | | 2 | 1 | |  | |  |
|  | AT06 | I don’t like to take latent TB infection treatment because it is unlikely that I will get latent TB infection | | 5 | 4 | | 3 | | 2 | 1 | |  | |  |
|  | AT07 | I am hesitant to take the medicine as I do not believe that I could possibly have latent TB | | 5 | 4 | | 3 | | 2 | 1 | |  | |  |
|  | AT08 | Latent TB infection treatment reduces TB incidence among PLHIV | | 1 | 2 | | 3 | | 4 | 5 | |  | |  |
|  | AT09 | Latent TB infection treatment is effective | | 1 | 2 | | 3 | | 4 | 5 | |  | |  |
|  | AT10 | Latent TB infection treatment is the key strategy to end TB | | 1 | 2 | | 3 | | 4 | 5 | |  | |  |
|  | AT11 | It is unsafe to give latent TB infection treatment to children and adolescents, and pregnant women | | 5 | 4 | | 3 | | 2 | 1 | |  | |  |
|  | AT12 | Latent TB infection treatment increases ART drug resistance | | 5 | 4 | | 3 | | 2 | 1 | |  | |  |
|  |  | **iv. PLHIV who have already initiated TPT: practice toward latent TB infection treatment** | | | | | | | |  | |  | |  |
|  | PR01 | Have you ever utilized the tracking table for medication used to treat latent tuberculosis infection, which is provided by health facilities? | | | | | Never  Sometimes  Always | | | | 0  1  2 |  | | |
|  | PRO2 | If yes, is the medication tracker table helpful? | | | | | Very helpful  Helpful  Intermediate  Not helpful  Not very helpful  Other(specify)_______ | | | | 0  1  2  3  4 |  | | |
|  | PR03 | If no, what would provide the most assistance for you to remember and adhere to your medication schedule? | | | | | SMS message from HCP  Cell phone alarm  Other(specify)_______ | | | | 0  1  2 |  | | |
|  | PR04 | Have you consistently marked/tapped the table while taking medication? | | | | | Never  Sometimes  Always | | | | 0  1  2 |  | | |
|  | PR05 | If response of PRO4 is never and sometimes, what can be a more effective prompt for you to remember taking your TPT medication than the medication tracker table provided by the healthcare facility? | | | | | Salat/pray time  Mealtime  Sleeping time  Other(specify)_______ | | | | 0  1  2  3 |  | | |
|  | PR06 | Have you ever forgotten to take your medication? | | | | | Never  Sometimes  Always | | | | 0  1  2 |  | | |
|  | PR07 | If you miss taking your medication for three consecutive days, will you resume taking it within the next three days and return to your regular schedule for taking it? | | | | | Never  Sometimes  Always | | | | 0  1  2 |  | | |
|  | PR08 | Which day would be the best for you to take your medication? | | | | | Sunday  Saturday  Monday  Tuesday  Wednesday  Thursday  Friday  NA: If the patient is not currently or previously receiving 3HP, choose 'NA' | | | | 0  1  2  3  4  5  6  7 |  | | |
|  | PR09 | What made you choose this day over others? | | | | | I have no any reason  It is not working day  it is associated with religion  Other(specify)_______ | | | | 0  1  2  3 |  | | |
|  | PR10 | If you completed all of your prescribed doses using the medication tracking chart, did you ever return the medication tracking chart to the HCP? | | | | | Never  Sometimes  Always | | | | 0  1  2 |  | | |
|  |  | **v. Implementation status of LTBI treatment: This part includes questions about screening and initiation of TPT up to TPT treatment outcome including M and E** | | | | | | | |  | |  | |  |
|  |  | **TPT implementation parameters** |  | | | |  | | |  | |  | |  |
|  | IS01 | **Screening and diagnosis** | Have you ever screened for latent TB infection at this healthcare facility? This indicates that your first contact for TB screening | | | | Yes  No | | | | 1  0 |  | | |
|  | IS02 |  | If yes, how many times have you screened? | | | | Once  Twice  Three times  Four times  Five times  Six and above times  I don’t remember | | | | 0  1  2  3  4  5  6 |  | | |
|  | IS03 |  | Outcome of screening | | | | Negative  Positive | | | | 0  1 |  | | |
|  | IS04 |  | Have you been screened for TB during each visit?  Chart review | | | | Yes  No | | | | 1  0 |  | | |
|  | IS05 |  | Have you ever been diagnosed with TB? Chart review | | | | Yes  No | | | | 1  0 |  | | |
|  | IS06 | **Monitoring and Evaluation** | At every follow-up appointment, were you screened for adverse events (AEs) related to the treatment while you were on TPT | | | | Yes  No | | | | 1  0 |  | | |
|  | IS07 |  | At follow-up visits during a TPT course, were you received standardized comprehensive education and counseling on TPT-related AEs | | | | Yes  No | | | | 1  0 |  | | |
|  | IS08 |  | Have you evaluated at the minimum interval of 2-4 weeks since initiating TPT? | | | | Yes  No | | | | 1  0 |  | | |
|  | IS09 |  | What is your current TPT status? | | | | On TPT  Completed  LTFU  Developed active  TB or Failed TPT  Others | | | | 0  1  2  3  4 |  | | |
|  | IS10 |  | Was the patient's CD4 count checked during every visit? | | | | Yes  No | | | | 1  0 |  | | |
|  | IS11 |  | Was the patient's viral load checked during every visit? | | | | Yes  No | | | | 1  0 |  | | |
|  | IS12 |  | Was the weight of the patient checked during every visit? | | | | Yes  No | | | | 1  0 |  | | |
|  | IS13 | **TPT initiation** | Was the patient screened for contraindications prior to starting TB Preventive Treatment? | | | | Yes  No | | | | 1  0 |  | | |
|  | IS14 |  | Have you monitored your TPT initiation? | | | | Yes  No | | | | 1  0 |  | | |
|  | IS15 |  | Did the patient start TPT medication right away after testing negative? | | | | Yes  No | | | | 1  0 |  | | |
|  | IS16 |  | Did the patient start medication with 3HP regimens? | | | | Yes  No | | | | 1  0 |  | | |
|  | IS17 | **TPT completion** | Have you monitored your TPT completion? | | | | Yes  No | | | | 1  0 |  | | |
|  | IS18 |  | Was the patient able to finish taking the medication within 12 weeks of starting? | | | | Yes  No | | | | 1  0 |  | | |
|  | IS19 |  | For how long you had/have been receiving TPT? | | | | >=1 week  2weeks  3weeks  4weeks  5weeks  6weeks  7weeks  8weeks  9weeks  10weeks  11weeks  12weeks  >12weeks  if >12 weeks, specify weeks | | | | 0  1  2  3  4  5  6  7  8  9  10  11  12 |  | | |
|  | IS20 |  | TPT outcome (Chart review_) | | | | Completed  LTFU  Died  Developed active  TB or Failed TPT  Not evaluated | | | | 0  1  2  3  4  5 |  | | |
|  | IS21 | **Adverse events management** | Have you ever experienced TB prevention treatment/therapy interruption or adverse events related to TB prevention? | | | | Yes  No | | | | 1  0 |  | | |
|  | IS22 |  | If yes to above question, who monitored your TPT interruption, and occurrence of adverse events related to TPT? | | | | HCP  Not monitored/No monitoring system in place  I don’t remember  Other, specify | | | | 0  1  2  3 |  | | |
|  | IS23 |  | Has the patient’s TPT regimen ever been changed due to side effects or new TB? | | | | Yes  No | | | | 1  0 |  | | |
|  | IS24 |  | Has the patient’s TPT regimen ever been stopped due to adverse events or diagnosed with active TB? | | | | Yes  No | | | | 1  0 |  | | |
|  |  | **vi. Health care providers related:** **This section addresses barriers that you may face in getting TPT service due to health care providers related factors. How would you rate the following as barriers that hinder you from getting TPT services? (Kindly tick the appropriate answers)** | | | | | | | |  | |  | |  |
|  |  |  | | | | **Not a barrier** | | **Somewhat a barrier** | | **Important barrier** | | |  |  |
|  | PF01 | Bad behaviour of health workers | | | | 1 | | 0 | | 0 | | |  |  |
|  | PF02 | Number availability of health care workers | | | | 1 | | 0 | | 0 | | |  |  |
|  | PF03 | Relationship with health provider | | | | 1 | | 0 | | 0 | | |  |  |
|  | PF04 | Being treated with respect | | | | 1 | | 0 | | 0 | | |  |  |
|  | PF05 | Experience of health provider | | | | 1 | | 0 | | 0 | | |  |  |
|  | PF06 | Attitude of health provider | | | | 1 | | 0 | | 0 | | |  |  |
|  | PF07 | No dedicated staff to lead this initiative | | | | 1 | | 0 | | 0 | | |  |  |
|  |  | Other(specify)_______ | | | |  | |  | |  | | |  |  |
|  |  | **vii. Institutional related factors: This section addresses the resources that are available to aid you in getting TPT services. A) Which of the following resources are available in your facility? This section addresses barriers that you may face in getting TPT service due to institutional related factors. How would you rate the following as barriers that hinder you from getting TPT services? (Kindly tick the appropriate answers)** | | | | | | | |  | | |  |  |
|  |  |  | | | | **Not a barrier** | | **Somewhat a barrier** | | **Important barrier** | | |  |  |
|  | IF01 | Lack of transport to health facility | | | | 1 | | 0 | | 0 | | |  |  |
|  | IF02 | Long distance to health facility | | | | 1 | | 0 | | 0 | | |  |  |
|  | IF03 | Lack drugs and supplies | | | | 1 | | 0 | | 0 | | |  |  |
|  | IF04 | Lack of privacy | | | | 1 | | 0 | | 0 | | |  |  |
|  | IF05 | Long waiting time at health facility | | | | 1 | | 0 | | 0 | | |  |  |
|  | IF06 | The hours of service at health facility, i.e., when they open and close | | | | 1 | | 0 | | 0 | | |  |  |
|  | IF07 | The cleanliness of the health facility | | | | 1 | | 0 | | 0 | | |  |  |
|  | IF08 | Lack of commination/coordination between TB/HIV program | | | | 1 | | 0 | | 0 | | |  |  |
|  | IF09 | Health facility officials' lack of commitment | | | | 1 | | 0 | | 0 | | |  |  |
|  |  | Other(specify)_______ | | | |  | |  | |  | | |  |  |

**Appendix S2.** English version MMAS-8.

| **Medication Adherence (Morisky 8-item questionnaire)** | | |
| --- | --- | --- |
|  | **Questions** | **Responses** |
| 1. | Do you sometimes forget to take your medication? | 0. Yes 1. No |
| 2. | People sometimes miss taking their medication for reasons other than forgetting. Over the past 2 weeks, were there any days you did not take your medicine? | 0. Yes 1. No |
| 3. | Have you ever cut back or stopped taking your medicine without telling your doctor because you felt worse when you took it? | 0. Yes 1. No |
| 4. | When you travel or leave home, do you sometimes forget to bring along your medicine? | 0. Yes 1. No |
| 5. | Did you take all your medicines yesterday? | 1. Yes 0. No |
| 6. | When you feel like your symptoms are under control, do you sometimes stop taking medicine? | 0. Yes 1. No |
| 7. | Taking medicine every day is a real inconvenience for some people. Do you ever feel hassled about sticking to your treatment plan? | 0. Yes 1. No |
| 8. | How often do you have difficulty remembering to take all your medicine? | 1. Never/rarely; 2. Occasionally;   3. Sometimes;  4. Usually;  5. All the time  1 = 1  2-5 = 0 |
